# The Epidemiological Influence of Climatic Factors on Shigellosis Incidence Rates in Korea

**DOI:** 10.3390/ijerph15102209

**Published:** 2018-10-10

**Authors:** Yeong-Jun Song, Hae-Kwan Cheong, Myung Ki, Ji-Yeon Shin, Seung-sik Hwang, Mira Park, Moran Ki, Jiseun Lim

**Affiliations:** 1 Department of Preventive Medicine College of Medicine, Eulji University, Daejeon 34824, Korea; syjace@nate.com (Y.-J.S.); mira@eulji.ac.kr (M.P.); 2 Department of Social and Preventive Medicine, Sungkyunkwan University School of Medicine, Suwon 16419, Korea; hkcheong@skku.edu; 3 Department of Preventive Medicine, College of Medicine, Korea University, Seoul 02841, Korea; myungki@korea.ac.kr; 4 Department of Preventive Medicine, School of Medicine, Kyungpook National University, Daegu 41944, Korea; jyshin@knu.ac.kr; 5 Department of Public Health Science, Graduate School of Public Health, Seoul National University, Seoul 08826, Korea; cyberdoc@snu.ac.kr; 6 Department of Cancer Control and Population Health, Graduate School of Cancer Science and Policy, National Cancer Center, Goyang 10408, Korea; moranki@ncc.re.kr

**Keywords:** meteorological factors, infectious diarrheal disease, shigellosis, seasonal variation

## Abstract

Research has shown the effects of climatic factors on shigellosis; however, no previous study has evaluated climatic effects in regions with a winter seasonality of shigellosis incidence. We examined the effects of temperature and precipitation on shigellosis incidence in Korea from 2002–2010. The incidence of shigellosis was calculated based on data from the Korean Center for Disease Control and Prevention (KCDC, Cheongju, Korea), and a generalized additive model (GAM) was used to analyze the associations between the incidence and climatic factors. The annual incidence rate of shigellosis was 7.9 cases/million persons from 2002–2010. During 2007–2010, high incidence rates and winter seasonality were observed among those aged ≥65 years, but not among lower age groups. Based on the GAM model, the incidence of shigellosis is expected to increase by 13.6% and 2.9% with a temperature increase of 1 °C and a lag of two weeks and with a mean precipitation increase of 1 mm and a lag of five weeks after adjustment for seasonality, respectively. This study suggests that the incidence of shigellosis will increase with global climate change despite the winter seasonality of shigellosis in Korea. Public health action is needed to prevent the increase of shigellosis incidence associated with climate variations.

## 1. Introduction

Shigellosis is an enteric infection caused by Gram-negative bacillus-shaped bacteria of the genus Shigella. The genus Shigella includes the species *Shigella dysenteriae*, *Shigella flexneri*, *Shigella boydii*, and *Shigella sonnei*. Symptoms of shigellosis include loose feces, fever, nausea, endotoxemia, vomiting, abdominal cramps, and tenesmus. Shigella bacteria are transmitted via direct or indirect fecal–oral routes from a symptomatic patient or a short-term asymptomatic carrier.

The World Health Organization classifies shigellosis as a waterborne and foodborne disease for which the development of a vaccine is imminent [1]. In the past 50 years, Shigella bacteria have developed resistance to numerous antibiotics and the global burden of shigellosis has increased worldwide [1,2]. It has recently been shown that 20% of hospitalized patients die of shigellosis; thus, developing a public health strategy for shigellosis disease management is critical [1,2,3].

In Korea, shigellosis is classified as a Group 1 nationally notifiable infectious disease because of the possibilities of shigellosis epidemics. *S. flexneri* and *S. sonnei* outbreaks occurred in Korea during the 1950–1980s and 1990–2000s, respectively [4]. In addition, recent studies have identified drug-resistant *S. sonnei* in Korea [5,6].

It has been predicted that there will be unprecedented global climate change that will lead to increases in waterborne and foodborne infectious diseases. Previous research has shown a positive association between temperature and shigellosis incidence; these studies were executed primarily in tropical and subtropical regions and showed a summer seasonality of shigellosis incidence [7,8,9,10,11,12,13,14,15,16]. The main transmission routes of shigellosis in Korea were known to be ingestion of contaminated water or food, as well as from person to person [17]. Recently, seasonal patterns of shigellosis in Korea have altered from spring/autumn to winter seasonality, indicating that the main transmission route or the vulnerable population may have changed in Korea [17,18]. Moreover, the effects of climate factors on shigellosis in Korea may differ from the results of previous studies due to the winter seasonality that occurs in Korea. This study was executed to evaluate the effect of temperature and precipitation on shigellosis incidence in Korea and to predict future trends based on global climate change. We also examined the effects of climatic factors across all four seasons to identify the seasons vulnerable to shigellosis incidence due to climate change.

## 2. Materials and Methods

### 2.1. Data Collection

The incidence of shigellosis from 2002–2010 was determined from nationally notifiable infectious disease data, which is managed by the surveillance division of the Korean Center for Disease Control and Prevention (KCDC, Cheongju, Korea). Infectious disease cases are reported by health providers working at hospitals or clinics to their regional health center, and the reports are transferred to the KCDC [19]. Finally, national statistics based on the reports are published after confirmation by the KCDC [19]. 

Raw climatic factor data were collected by the automatic weather system (AWS) of the Korean Meteorological Administration (KMA, Seoul, Korea) from 2002–2010. The KMA operates 494 AWSs all over the country to provide real-time weather information, and raw data is collected at 10 min intervals [20]. The climatic data were processed by region every week based on structured grid data with 1 km resolution [20,21]. Population data from 2002–2010 were obtained from the resident registration population report by Statistics Korea (Daejeon, Korea). This research was conducted in accordance with the Declaration of Helsinki, and the protocol was approved by the Institutional Review Board (IRB No. EU-14-06) of Eulji Medical University (Daejeon, Korea).

### 2.2. Statistical Analysis

The incidence rates for each sex and age group were determined and age-standardized rates were calculated using Microsoft Excel 2010 (Microsoft, Redmond, WA, USA). The population number in 2006 was used as the standard population when calculating the age-standardized rate. The age groups were categorized as 0–2 years (infant), 3–6 years (child), 7–17 years (juvenile), 18–64 years (adult), and 65 years and over (elderly) [22]. 

We compared seasonal patterns and age-specific incidence rates between 2002–2006 and 2007–2010 because shigellosis incidence and seasonal patterns changed from 2007. A generalized additive model (GAM) was used to evaluate linear and nonlinear associations of shigellosis incidence with temperature and precipitation, respectively. The unit of analysis was province, and datasets were constructed across seven metropolitan cities and nine provinces.

Penalized thin plate regression splines and logarithm link functions were applied to the GAM. We considered shigellosis incidence as a quasi-Poisson distribution because the scale estimate was calculated to be ≥10 [23]. Model selection was based on the lowest generalized cross validation score and the highest deviance explained value. Model selection was based on the lowest generalized cross validation score and the highest deviance explained value. Equations (1) and (2) were used to estimate the linear effects of temperature and precipitation, respectively. Equation (3) was used to describe the nonlinear effects of temperature and precipitation. In Equation (4), the upper limit on the degrees of freedom of each week was divided according to the four seasons of Korea.

- Equation (1). GAM for evaluating the effect of temperature
  (1) $$\begin{array}{c} g\left(E\left(Y\right)\right)=\alpha +offset\left(log\left(population\right)\right)+\beta_{1}\left(temperature_{i}\right)+s_{1}\left(precipitation_{i}\right)\\ +s_{2}\left(week_{i},\;df=53\right)+s_{3}\left(year_{i},\;df=9\right) \end{array}$$
- Equation (2). GAM for evaluating the effect of precipitation
  (2) $$\begin{array}{c} g\left(E\left(Y\right)\right)=\alpha +offset\left(log\left(population\right)\right)+s_{1}\left(temperature_{i}\right)+\beta_{1}\left(precipitation_{i}\right)\\ +s_{2}\left(week_{i},\;df=53\right)+s_{3}\left(year_{i},\;df=9\right) \end{array}$$
- Equation (3). GAM for smoothing plots
  (3) $$\begin{array}{c} g\left(E\left(Y\right)\right)=\alpha +offset\left(log\left(population\right)\right)+s_{1}\left(temperature_{i}\right)+s_{2}\left(precipitation_{i}\right)\\ +s_{3}\left(week_{i},\;df=53\right)+s_{4}\left(year_{i},\;df=9\right) \end{array}$$
- Equation (4). GAM for seasonality stratification
  (4) $$\begin{array}{c} g\left(E\left(Y\right)\right)=\alpha +offset\left(log\left(population\right)\right)+s_{1}\left(temperature_{i}\right)+s_{2}\left(precipitation_{i}\right)\\ +s_{3}\left(week_{i},\;df=t\right)+s_{4}\left(year_{i},\;df=9\right) \end{array}$$

E(Y) is the expected number of shigellosis cases, $temperature_{i}$ is the weekly average of the daily peak temperature, $precipitation_{i}$ is the weekly average of daily precipitation, ***population*** is the population number in the province, $week_{i}$ and $year_{i}$ are the corresponding periods of incidence, ***α*** is the dummy variable for the incidence of shigellosis, ***df*** is the upper limit on the degrees of freedom, and t is the number of seasonal week. The actual effective degrees of freedom are automatically corrected by the degree of penalization selected during fitting. An offset term was used to adjust for population size. The $temperature_{i}$, $precipitation_{i}$, $week_{i}$, and $year_{i}$ were adjusted with spline function s for smoothing.

The lag time between the change in climatic factors and the incidence of shigellosis was set from 0–6 weeks, including the time required for Shigella growth, contamination of water or food, occurrence of the intestinal infection, diagnosis of the infection, and notification of the shigellosis incident [24,25]. Further, to investigate the vulnerable season due to changes in climatic factors, a stratified association analysis was performed for all four seasons. The GAM analysis was conducted with “mgcv,” “season,” and “Hmis” packages using the “gam” command in R-3.2.0 for Windows (R Foundation for Statistical Computing, Vienna, Austria).

## 3. Results

### 3.1. Distribution of Shigellosis Incidence across the Seasons according to Age

**Figure 1:**
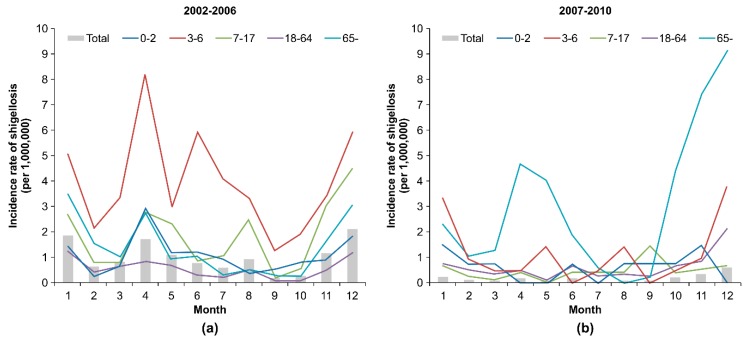
Annual incidence rate (per million) of shigellosis by age, 2002–2010.

The annual average incidence rate of shigellosis from 2002–2010 was 7.9 cases per 1,000,000 persons. The annual incidence rate was the highest in 2003 with 23.0 cases per 1,000,000 persons and it gradually declined after 2006. The incidence rate was higher among women than men in every year. The incidence rates decreased significantly after 2006 for all of the age groups except the elderly. Additionally, from 2008–2010, the incidence rate was twice as high for the elderly (11.5, 9.9, and 8.0 cases per 1,000,000 per year, respectively) than for children (4.1, 3.2, 3.8 cases per 1,000,000 per year, respectively) and four times higher than for the other age groups (Table 1). From 2002–2006, the incidence of shigellosis showed spring and winter seasonality across most of the age groups. From 2007–2010, the incidence of shigellosis showed winter seasonality, especially among the elderly (Figure 1).

### 3.2. Association between Climatic Factors and the Incidence of Shigellosis

The incidence rate of shigellosis showed positive associations with temperature and precipitation at all lag times. The associations of incidence of shigellosis with temperature (lag week: 0–6) and precipitation (lag week: 0, 4–6) were statistically significant. A 1 °C increase in temperature and a 1 mm increase in precipitation were associated with a 13.6% (95% confidence interval (CI) 9.2–18.0%) and a 2.9% (95% CI: 0.5–5.2%) maximum increase in shigellosis incidence after two-week and five-week lags, respectively (Table 2).

**Figure 2:**
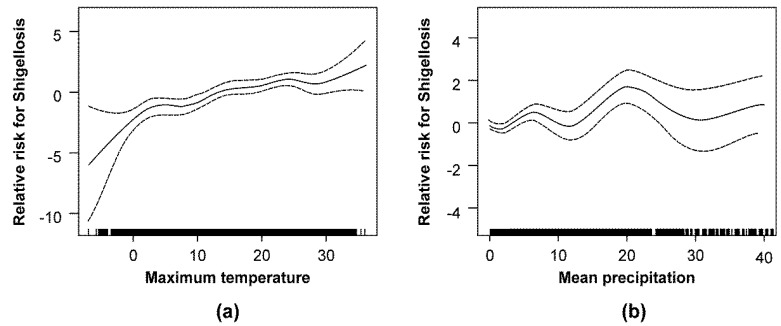
Smoothing plots of (**a**) the weekly maximum temperature and (**b**) mean precipitation and their relationships to shigellosis in Korea, 2002–2010. Note: The continuous line indicates the relative risk for shigellosis. The regions above and below the discontinuous lines indicate the confidence interval of the relative risk for shigellosis.

There was an overall positive association between temperature and the shigellosis incidence rate. The degree of the association was larger below 4 °C than above it, although the precision of relative risk was lower below 4 °C than above it; the lower the temperature below 4 °C was, the wider the 95% confidence interval of relative risk was as observed in Figure 2. In contrast, the risk of shigellosis showed a nonlinear waxing and waning pattern when its association with increases in precipitation was evaluated (Figure 2).

The associations between the incidence rates of shigellosis and temperature and precipitation differed for each season. In the spring, a 1 °C increase in temperature and a 1 mm increase in precipitation were associated with a 16.1% (95% CI: 7.8–24.5%) and an 8.4% (95% CI: 4.7–12.1%) maximum increase in shigellosis incidence after a two-week lag, respectively. In summer, a 1 °C increase in temperature and a 1 mm increase in precipitation were associated with a maximum 17.5% (95% CI: 3.2–31.7%) increase and a maximum 2.9% (95% CI: 0.1–5.7%) decrease in shigellosis incidence at one-week and two-week lags, respectively. In autumn, a 1 °C increase in temperature and a 1 mm increase in precipitation were associated with a 20.0% (95% CI: 8.0–32.0%) and a 11.8% (95% CI: 3.1–20.4%) maximum increase in shigellosis incidence at two-week and zero-week lags, respectively. In winter, a 1 °C increase in temperature and a 1 mm increase in precipitation were associated with a 17.4% (95% CI: 12.6–22.2%) increase and a 15.3% (95% CI: 4.2–26.4%) decrease in shigellosis incidence after two-week and zero-week lags, respectively (Table 3).

## 4. Discussion

This is the first study to confirm the association of climatic factors with shigellosis in a region that has shown winter seasonality for shigellosis. The shigellosis incidence rate in Korea associated positively with temperature and precipitation at all of the lag times. After stratification by season, the effect of temperature was prominent, whereas the effect of precipitation was variable.

The reduction in shigellosis incidence among children and juveniles may be attributed to active public hygiene interventions for daycare and food service facility workers since 2006 [26,27,28,29,30,31,32]. In contrast, the incidence of shigellosis among the elderly was consistent in 2007–2010. Introduction of National Long-Term Care Insurance for the elderly in July 2008 led to a drastic increase in the number of elderly care facilities and their residents, which may have led to increased transmission of healthcare-associated infections in elderly care facilities [33,34]. Moreover, the elderly population was not included as a priority target for national sanitation control. Consequently, these factors may have caused the high incidence rate of shigellosis among the elderly during the late 2000s [35,36]. 

There have been limited studies on winter patterns of shigellosis seasonality, and they have failed to clearly identify the mechanism underlying these winter patterns [37,38]. From 1978–1988, winter patterns of shigellosis seasonality were observed in Milwaukee, United States and were attributed to the lack of appropriate sanitary control in daycare centers in major cities during the winter [38]. One possible reason for the winter pattern of seasonality observed among the elderly in this study is the characteristic behavior of rural elderly people to spend long periods of time together in senior citizen centers or community halls during the winter [39]. 

In this study, the estimated effect of rises in the weekly average of the daily peak temperature and the weekly average of the daily precipitation was an increase in the incidence rate of shigellosis. Previous studies from tropical and subtropical regions of China and Vietnam also reported a similar effect of temperature on shigellosis [8,12,14,15,16]. The estimated positive effect of precipitation on shigellosis in our study was concordant with results from some studies [11,12,15,16], but inconsistent with results from other studies [13]. Further studies are needed to estimate the effects of climatic factors on shigellosis incidence in temperate regions.

According to the smoothing plot, the risk of shigellosis increased linearly with increases in temperature, whereas the risk of shigellosis waxed and waned with increases in precipitation yet displayed an overall increase. The larger association between temperature and shigellosis incidence below 4 °C observed in the smoothing plot suggests that a 1 °C increase in temperature may cause a greater increase of shigellosis incidence in cold temperatures than in warm temperatures. A previous study that used GAM modeling showed a positive linear association of shigellosis with temperature and a fluctuating nonlinear association of shigellosis with precipitation, which is consistent with our results [14]. The waxing and waning pattern of shigellosis risk with increases in precipitation can be explained. Contamination of drinking water due to heavy rainfall can increase the risk of shigellosis and can explain the positive association between precipitation and shigellosis incidence. In addition, relative humidity is generally higher with high precipitation, and high relative humidity can shorten the survival period of bacteria within the normal temperature interval of growth and can explain the negative association between precipitation and shigellosis incidence [40]. The positive effect of temperature on shigellosis incidence was consistent across all four seasons. In contrast, the effect of precipitation was negative during summer and winter, which may be the result of the complex association between precipitation and shigellosis incidence.

This study had several limitations. First, although various factors, such as the ecology of the infectious agent and the behavior of the population, may affect the incidence of shigellosis, we only considered the influence of climatic factors on the incidence of shigellosis. Future studies should include other factors, such as humidity and the behavior of the population, which may affect the incidence of shigellosis. Second, because nationally notifiable infectious diseases are reported by health care providers, the statistics do not reflect all cases of infections. Although the reporting rate is lower than the actual incidence rate, it cannot be concluded that the level of underreporting was affected by the climatic factors, and this causes non-differential misclassification. If all cases had been reported, the associations may have been higher than observed in this study. Although the reporting rate of sporadic infections is usually low, the reporting rate is higher if infections occur in educational or daycare facilities where investigations of epidemics are conducted regularly. Differences in reporting rates, which vary according to emerging patterns of infections, may have affected this analysis on the association between shigellosis and climatic factors.

This study was meaningful in that it was the first to analyze the association between the incidence of shigellosis and climatic factors in the Korean population and to perform a stratified analysis to identify vulnerable seasons. Second, the study revealed that the winter patterns of seasonality that have been observed since the late 2000s occurred primarily among the elderly population; thus, we recommend enforcement of sanitary control in this population.

## 5. Conclusions

In this study, the incidence of shigellosis in Korea associated positively with temperature and precipitation, and we predict that the incidence of shigellosis will increase with global climate changes in the future. Therefore, consistent and careful monitoring of the incidence of shigellosis is necessary from a public health perspective. Moreover, further analyses on the association of shigellosis with climate and identification of populations vulnerable to climate change in Korea are necessary.

## Figures and Tables

**Table 1:** Annual incidence rate (per million) of shigellosis by sex and age, 2002–2010.

Year	2002	2003	2004	2005	2006	2007	2008	2009	2010	Total
Total	15.73	23.00	9.57	5.61	7.55	1.83	2.98	2.92	2.49	7.90
Sex										
Men	13.46	22.36	9.54	5.00	6.94	1.50	2.67	1.77	1.92	7.18
Women	18.02	23.64	9.60	6.22	8.16	2.16	3.29	4.08	3.05	8.62
Age (years)										
0–2	15.04	26.99	5.42	7.11	8.87	0.00	3.66	2.17	1.46	8.37
3–6	56.59	113.55	12.72	13.83	36.90	3.80	4.09	3.21	3.79	30.84
7–17	24.36	45.10	28.44	4.83	8.18	0.94	2.56	1.51	0.85	13.02
18–64	9.52	10.11	5.11	3.97	5.24	1.52	1.72	2.18	1.93	4.55
≥65	24.56	28.09	10.96	14.68	8.56	5.10	11.48	9.87	8.04	12.78

**Table 2:** Associations between shigellosis incidence and climatic factors.

Climatic Factors	Time-Point	Relative Risk	95% Confidence Interval		Change (%)	*p*-Value
Maximum temperature	Present	1.100	1.060	1.140	10.0	<0.001
	Lag 1	1.128	1.085	1.171	12.8	<0.001
	Lag 2	1.136	1.092	1.180	13.6	<0.001
	Lag 3	1.106	1.063	1.149	10.6	<0.001
	Lag 4	1.098	1.057	1.139	9.8	<0.001
	Lag 5	1.076	1.033	1.119	7.6	<0.001
	Lag 6	1.080	1.038	1.122	8.0	<0.001
Daily precipitation	Present	1.025	1.003	1.047	2.5	0.030
	Lag 1	1.021	0.997	1.045	2.1	0.094
	Lag 2	1.020	0.996	1.045	2.0	0.110
	Lag 3	1.012	0.987	1.038	1.2	0.342
	Lag 4	1.026	1.003	1.049	2.6	0.026
	Lag 5	1.029	1.005	1.052	2.9	0.018
	Lag 6	1.029	1.004	1.054	2.9	0.026

Note: Statistical analyses were conducted using the generalized additive model and seasonality was corrected by spline functions. When the effect of temperature was primarily examined, corrections for precipitation were made, and when the effect of precipitation was primarily examined, corrections for temperature were made.

**Table 3:** Associations between shigellosis incidence and climatic factors after seasonal stratification.

Season	Time-Point	Maximum Temperature					Mean Precipitation				
		RR	95% CI		Change (%)	*p*-Value	RR	95% CI		Change (%)	*p*-Value
Spring	Present	1.047	0.968	1.126	4.7	0.252	1.049	1.017	1.081	4.9	0.003
	Lag 1	1.044	0.969	1.119	4.4	0.259	1.046	1.010	1.083	4.6	0.016
	Lag 2	1.161	1.078	1.245	16.1	<0.001	1.084	1.047	1.121	8.4	<0.001
	Lag 3	1.122	1.050	1.194	12.2	0.002	1.082	1.032	1.133	8.2	0.002
	Lag 4	1.070	0.999	1.141	7.0	0.063	1.037	0.975	1.099	3.7	0.252
	Lag 5	1.023	0.949	1.098	2.3	0.544	1.041	0.979	1.103	4.1	0.206
	Lag 6	1.048	0.973	1.123	4.8	0.224	1.066	0.996	1.135	6.6	0.072
Summer	Present	0.997	0.877	1.116	−0.3	0.955	1.000	0.978	1.022	0.0	0.989
	Lag 1	1.175	1.032	1.317	17.5	0.027	0.999	0.971	1.027	−0.1	0.939
	Lag 2	1.040	0.904	1.175	4.0	0.574	0.971	0.943	0.999	−2.9	0.039
	Lag 3	0.968	0.848	1.088	−3.2	0.592	0.981	0.956	1.005	−1.9	0.116
	Lag 4	1.009	0.900	1.117	0.9	0.875	1.015	0.995	1.035	1.5	0.149
	Lag 5	0.887	0.780	0.995	−11.3	0.029	1.001	0.981	1.021	0.1	0.905
	Lag 6	0.967	0.857	1.077	−3.3	0.554	1.011	0.988	1.035	1.1	0.354
Autumn	Present	1.067	0.974	1.160	6.7	0.174	1.118	1.031	1.204	11.8	0.012
	Lag 1	1.186	1.073	1.300	18.6	0.003	1.066	0.982	1.149	6.6	0.134
	Lag 2	1.200	1.080	1.320	20.0	0.003	1.027	0.935	1.118	2.7	0.573
	Lag 3	1.179	1.061	1.297	17.9	0.006	0.994	0.906	1.082	−0.6	0.892
	Lag 4	1.177	1.048	1.306	17.7	0.013	1.069	1.011	1.126	6.9	0.024
	Lag 5	1.183	1.041	1.324	18.3	0.020	1.030	0.974	1.087	3.0	0.301
	Lag 6	1.149	1.006	1.292	14.9	0.058	1.007	0.956	1.058	0.7	0.785
Winter	Present	1.098	1.051	1.145	9.8	<0.001	1.071	0.977	1.165	7.1	0.152
	Lag 1	1.156	1.108	1.205	15.6	<0.001	1.074	0.974	1.175	7.4	0.163
	Lag 2	1.167	1.120	1.214	16.7	<0.001	1.018	0.917	1.119	1.8	0.727
	Lag 3	1.122	1.075	1.169	12.2	<0.001	1.039	0.939	1.140	3.9	0.453
	Lag 4	1.174	1.126	1.222	17.4	<0.001	0.847	0.736	0.958	−15.3	0.003
	Lag 5	1.138	1.085	1.191	13.8	<0.001	0.982	0.880	1.085	−1.8	0.735
	Lag 6	1.108	1.059	1.157	10.8	<0.001	0.991	0.888	1.093	−0.9	0.856

Note: RR: relative risk; 95% CI: 95% confidence interval. Statistical analyses were conducted using the generalized additive model and seasonality was corrected by spline functions. When the effect of temperature was primarily examined, corrections for precipitation were made, and when the effect of precipitation was primarily examined, corrections for temperature were made.
